# Combination of aquifer thermal energy storage and enhanced bioremediation: resilience of reductive dechlorination to redox changes

**DOI:** 10.1007/s00253-015-7241-6

**Published:** 2015-12-28

**Authors:** Zhuobiao Ni, Pauline van Gaans, Martijn Smit, Huub Rijnaarts, Tim Grotenhuis

**Affiliations:** Sub-Department of Environmental Technology, Wageningen University, P.O. Box 17, 6700 AA Wageningen, the Netherlands; Wetsus, European Centre of Excellence for Sustainable Water Technology, P.O. Box 1113, 8900 CC Leeuwarden, the Netherlands; Soil and Groundwater Systems, Deltares, P.O. Box 85467, 3508 AL Utrecht, the Netherlands; Eurofins Analytico, P.O. Box 459, 3770 AL Barneveld, the Netherlands

**Keywords:** Reductive dechlorination, Aquifer thermal energy storage (ATES), *cis*-dichloroethene (*cis*-DCE), *Dehalococcoides*, Microbial resilience, Redox potential (*E*_Ag/AgCl_)

## Abstract

To meet the demand for sustainable energy, aquifer thermal energy storage (ATES) is widely used in the subsurface in urban areas. However, contamination of groundwater, especially with chlorinated volatile organic compounds (CVOCs), is often being encountered. This is commonly seen as an impediment to ATES implementation, although more recently, combining ATES and enhanced bioremediation of CVOCs has been proposed. Issues to be addressed are the high water flow velocities and potential periodic redox fluctuation that accompany ATES. A column study was performed, at a high water flow velocity of 2 m/h, simulating possible changes in subsurface redox conditions due to ATES operation by serial additions of lactate and nitrate. The impacts of redox changes on reductive dechlorination as well as the microbial response of *Dehalococcoides* (DHC) were evaluated. The results showed that, upon lactate addition, reductive dechlorination proceeded well and complete dechlorination from *cis*-DCE to ethene was achieved. Upon subsequent nitrate addition, reductive dechlorination immediately ceased. Disruption of microorganisms’ retention was also immediate and possibly detached DHC which preferred attaching to the soil matrix under biostimulation conditions. Initially, recovery of dechlorination was possible but required bioaugmentation and nutrient amendment in addition to lactate dosing. Repeated interruption of dechlorination and DHC activity by nitrate dosing appeared to be less easily reversible requiring more efforts for regenerating dechlorination. Overall, our results indicate that the microbial resilience of DHC in biosimulated ATES conditions is sensitive to redox fluctuations. Hence, combining ATES with bioremediation requires dedicated operation and monitoring on the aquifer geochemical conditions.

**Electronic supplementary material:**

The online version of this article (doi:10.1007/s00253-015-7241-6) contains supplementary material, which is available to authorized users.

## Introduction

Aquifer thermal energy storage (ATES) has been developed since the 1970s and is considered as an energy saving and sustainable energy technology (Hendriks et al. 2008; Lee 2013; Novo et al. 2010). The principle of ATES is to store thermal energy when available and retrieve it for use when needed. In summer, groundwater is extracted from ATES cold well. At the heat exchanger, the excessive heat from the building is transported to this water which is then re-injected into the aquifer at the ATES warm well. In the aquifer, a large fraction of heat remains available to be used in winter when the groundwater flow and energy extraction process will be reversed (Lee 2013; Paksoy 2007). Due to an increased demand for sustainable energy, existing ambitions to reduce greenhouse gas emission and its cost-effectiveness, ATES is increasingly being developed throughout the world, especially in urban areas (EC 2007; Lee 2010; Snijders 2005; Sommer et al. 2015). For ATES development, Belgium, Germany, the Netherlands, Turkey, Sweden and the USA are currently leading (Andersson et al. 2013; Paksoy et al. 2004; Paksoy et al. 2009; Paksoy 2007; Sanner et al. 2005; Vanhoudt et al. 2011). For example, the number of ATES systems was 2740 in the Netherlands (CBS 2010) and is expected to rise to 20,000 in 2020 (Godschalk and Bakema 2009). Meanwhile, many urban areas are confronted with groundwater contaminations in the aquifer layer, especially with chlorinated solvents (Burston et al. 1993; Lopes and Bender 1998; Moran et al. 2007; Squillace et al. 2002). Chlorinated solvents, in particular, tetrachloroethene (PCE) and trichloroethene (TCE), have been applied widely as degreasers in industrial factories and for dry-cleaning since the 1930s (ITRC 2005; Linn et al. 2004; Longstaff et al. 1992). Due to improper disposal, leakage and accidents, PCE and TCE together with their daughter products *cis*-dichloroethene (*cis*-DCE) and vinyl chloride (VC) have become the most prevalent groundwater contaminants in aquifers (Grindstaff 1998; Henry and Warner 2003). These chlorinated volatile organic compounds (CVOCs) are often found at a depth in the subsurface similar to that at which ATES is usually applied (McCarty 2010; Zuurbier et al. 2013). ATES involves the transportation of large volumes of groundwater, for instance 261 million m^3^ groundwater was displaced by ATES in the Netherlands in 2013(Verburg et al. 2010). Hence, interference between CVOCs and ATES application at the same site can be a threat not only to the quality of groundwater and drinking water but also to human health due to the vapour intrusion especially of the carcinogenic intermediate VC (Picone 2012). Faced with the long duration commonly needed for natural attenuation of CVOCs in the subsurface (ITRC 2008; Wilson et al. 2007) and the increasing demand for ATES, the combination of ATES and stimulated bioremediation might be prosperous to reduce the increasing pressure on the use of the subsurface. The increased temperature around the ATES warm well (20–25 °C) (Slenders et al. 2010; Zuurbier et al. 2013) and using ATES as biostimulation tool, for example, for addition of electron donor, are the two main positive perspectives recognized for such combination (Sommer 2015). Currently, two field scale tests are being performed in the Netherlands where ATES is applied at CVOCs contaminated sites (Lieten et al. 2012; Slenders et al. 2010), which so far have not encountered practical problems. Furthermore, our previous laboratory study under simulated ATES conditions, focusing on *cis*-DCE biodegradation, showed 13 times higher degradation capacity at increased temperature compared to biodegradation by enhanced natural attenuation at groundwater temperature of 10 °C (Ni et al. 2015). These findings are promising; however, the impact on the microbial population is unclear, especially regarding the *Dehalococcoides* (DHC) species as the only group of bacteria strains capable of completely dechlorinating CVOCs to the harmless product ethene (Aulenta et al. 2006; Ballapragada et al. 1997; Boopathy 2000; Maymó-Gatell et al. 2001; McCarty 1997; van der Zaan et al. 2010).

The displacement of large volumes of groundwater by the ATES system will also have an impact on the local geochemical state of the aquifer. Redox conditions (of groundwater) in natural (undisturbed) aquifers can show a heterogeneous pattern over different depths and lateral positions (Christensen et al. 2000; McMahon and Chapelle 2008). The mixing of large volumes of groundwater, together with temperature changes, may then result in an overall change of redox condition on the re-injected groundwater and thus the aquifer zone around the injection well (Bonte et al. 2013; Jesußek et al. 2013; Possemiers et al. 2014). Because of the seasonal operation of ATES, such change of redox condition can occur in both warm and cold zones. In the worst case, redox change can lead to well clogging by precipitation of iron (hydr)oxides when, for example, dissolved iron encounters nitrates or dissolved oxygen (Brown and Misut 2010; Houben 2006; Kohfahl et al. 2009; Possemiers 2014; van Beek 2011). Also, for microbiological processes in the combination of ATES and bioremediation of CVOCs, the redox potential is a crucial factor, as DHC, which is primarily involved in the reductive dechlorination process, is sensitive to the redox condition (Amos et al. 2008; Maymó-Gatell et al. 2001; Smidt and de Vos 2004; van der Zaan et al. 2010) and especially vulnerable to oxidized redox condition such as nitrate reducing and oxidizing condition (Nobre and Nobre 2004). Exposure to air or oxygen was found to cause irreversible inhibition of reductive dechlorination (Amos et al. 2008). Several studies have revealed that DHC is natively found in many contaminated sites, though often at a very low number (Lendvay et al. 2003; Major et al. 2002). Moreover, DHC is more abundant at contaminated sites with lower redox potential, where reductive dechlorination is actively occurring (Behrens et al. 2008; Lu et al. 2006; Schaefer et al. 2009). Therefore, changing redox condition of groundwater by ATES operation is expected to influence the CVOCs reductive dechlorination by affecting the activity of DHC. Previous studies showed the inhibition effect of nitrate on the dechlorination process of chloroaliphatic (Gerritse et al. 1997; Keith et al. 2005; Mohn and Tiedje 1992; Nelson et al. 2002; Picardal et al. 1995; Ritter et al. 2003; Semprini et al. 1992) and chloroaromatic compounds (Chang et al. 1996; Chen et al. 2002; Genthner et al. 1989; Häggblom et al. 1993; Middeldorp et al. 2005; Milligan and Häggblom 1999; Okutman Tas and Pavlostathis 2008), while only very few studies focused on the responsible microorganism DHC upon its reaction to oxygen (Adrian et al. 2007; Amos et al. 2007; Amos et al. 2008). However, these studies have not covered the situation of possible variation of redox conditions due to seasonal operation of ATES. Considering the need of both sustainable energy production and more effective remediation of groundwater contaminants, especially in urban areas, it is wise to investigate the impacts of ATES on bioremediation of these contaminants in advance. Therefore, as the prevalent groundwater contaminants, biodegradation of CVOCs was studied under a simulated ATES condition in this study. We aim to improve the understanding of the resilience of the dechlorinating process by bacteria that are active in contaminated aquifers upon disturbances by redox fluctuation that can be generated by the functioning of an ATES system. We focus on the warm well as we expect that most of the conversion of CVOCs is located at that area. Dynamic redox conditions that might occur in an ATES system were simulated at lab scale by consequent nitrate and lactate additions and were used to determine chemical changes as well as effects on the microbial community and its characteristics.

## Materials and methods

### Materials

Aquifer material from the city of Utrecht, similar as in our previous study (Ni et al. 2014) on PCE biodegradation in a Fe(III) reducing aquifer, was used. All chemical stock solutions were prepared with anaerobic deionized water, which was purged with pure N_2_ for at least 3 h. Sodium lactate (SL) stock solution (225 g/L), *cis*-DCE stock solution (395 mg/L) and NaNO_3_ stock solution (85 g/L) were prepared, respectively, with SL powder (≥99 % purity, Aldrich®), NaNO_3_ powder (≥99 % purity, Aldrich®) and *cis*-DCE (99 % anhydrous, Aldrich®). Anaerobic tap water, prepared similarly as anaerobic deionized water, from the lab was used as liquid medium in the column experiment.

A mixed culture containing 1.3–4.4 × 10^8^ DHC cells/mL liquid (or DNA copies/mL liquid, based on the assumption that 1 DNA-copy is equal to 1 cell) was provided by Bioclear BV (Groningen, NL). This culture was occasionally fed with *cis*-DCE and lactate prior to the experiment. Inoculation was performed when *cis*-DCE and VC were found absent in the headspace of the inoculum bottle.

### Analytical methods

Redox potential and pH of the experimental systems were monitored by a Consort multi-channel (C3060) metre and data logger with ProSense (QIS) standard Pt redox electrode with Ag/AgCl as reference electrode (−199 mV vs. standard hydrogen electrode (SHE)) in saturated KCl solution (Bard and Faulkner 2001) and standard pH electrode (QIS), respectively. Temperature was also monitored and logged by C3060 metre. Logging interval of redox potential, pH and temperature was 1 min.

Dissolved Fe^2+^, SO_4_^2−^, lactate and volatile fatty acids (VFAs) were determined using the same analytical methods as in our previous study with redox titration method on PCE biodegradation in Fe(III) reducing aquifer (Ni et al. 2014). Biogas was analysed and quantified for CO_2_, CH_4_ and O_2_ in the headspace on a Shimadzu 2010 GC with thermal conductivity detector (TCD) and with helium as carrier gas. Loop injection of 2 mL headspace sample was performed at 120 °C. Chlorinated ethene (*cis-*DCE and VC) and ethene were quantified by direct injection of 100 μL (with glass syringe) on a HP6890 series GC equipped with a CP PoraBond Q column (25 m × 0.53 mm × 10 μm) and flame ionization detector (FID). Nitrogen gas was used as carrier gas at a flow rate of 25 mL/min. The temperature programme started at 60 °C, ramped at 17.78 °C/min to 140 °C and held at 140 °C for 1.5 min.

Microbial analysis (total bacteria and DHC cell^*^) was done by BioClear BV. Before analyses, 5 mL effluent from the column was collected and stored at 4 °C, in a special storage kit from Bioclear BV.

### Recirculating column set-up

#### Overview and specification

**Fig. 1:**
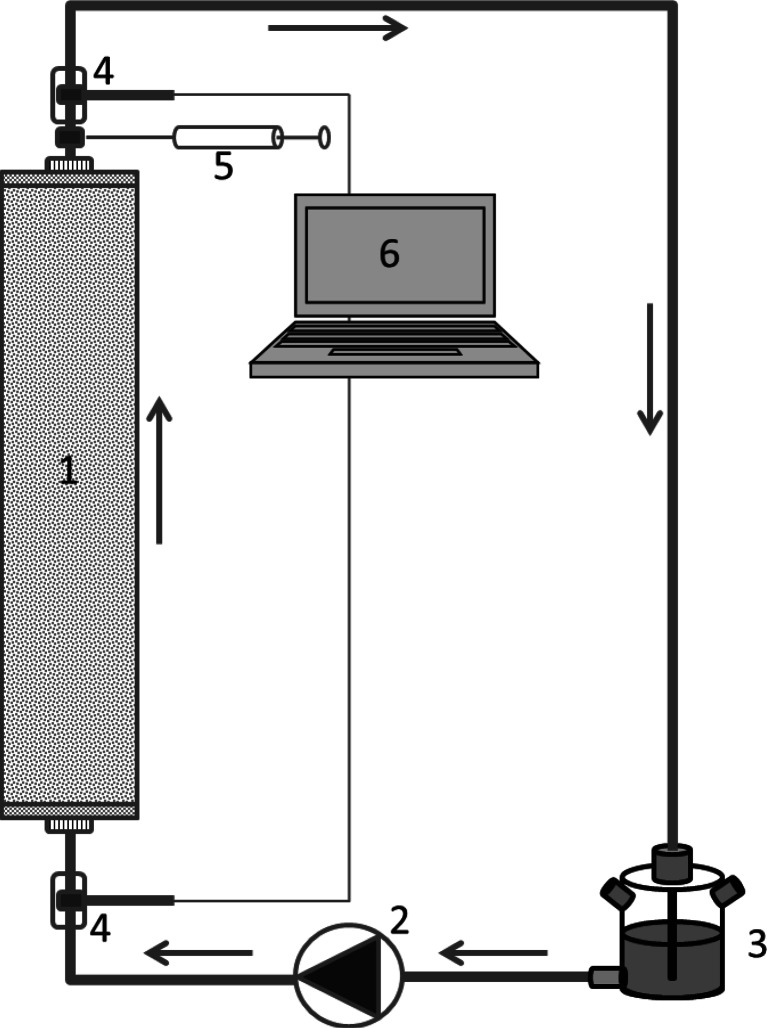
Schematic diagram of column set-up. *1* aquifer material with glass filters with pore size of 160 μm on the top and bottom of the column; *2* membrane pump; *3* buffering bottle; *4* redox and pH electrodes (*bottom* is for influent, *top* is for effluent); *5* liquid sampling port; *6* metre connected with computer. *Arrow* indicates the direction of water flow

The schematic overview and specification of the column set-up are given in Fig. 1, Table S1 and Figure S1. The various parts of the column set-up were connected by Teflon tubings (∅4 mm) with quick connectors with valves. The aqueous phase was recirculated by a SIMDOS® diaphragm metering pump (flow rate range: 1–100 mL/min). Glass filters (pore size with P2 specification: 100–160 μm) were placed on the top and bottom of the column to prevent particles from being flushed out. A 150-mL double-side armed bottle with approximately 100-mL headspace volume was placed in the middle of the system for headspace sampling and to prevent under-pressure during liquid and headspace sampling.

#### Preparation and maintenance of column

All individual parts of the column setup were filled inside an anaerobic hood (95 % N_2_ and 5 % H_2_) with either aquifer material or anaerobic tap water. The buffering bottle was additionally conditioned by exchanging the headspace 10 times with 99 % N_2_ and 1 % CO_2_ gas by an automatic headspace exchanger. The individual parts of the set-up were then attached by the quick connectors and, after opening the valves, the aqueous phase was allowed to circulate at a flow rate of 10 mL/min. This flow rate is comparable to a groundwater velocity of 2 m/h, which is a realistic flow velocity in aquifers 1.3 m away from an ATES well. This ATES system is assumed to operate with a flow rate of 100 m^3^/h and with 20 m filter length (see calculation in Figure S2).

During the first few days, the liquid level in the buffering bottles was lowered due to degassing of the aquifer column and tubing. Extra anaerobic tap water was injected in the bottle to maintain the liquid volume at approximately 50 mL. This procedure to maintain the liquid volume was also carried out later during the experiment, in case more gas bubbles were produced by degassing from the column. Moreover, after every liquid sampling, the same amount of anaerobic tap water as liquid sample was injected.

During the whole experimental period, small but continuous diffusion of oxygen was occurring, resulting in a volume concentration of about 1 % in the headspace of the buffering bottle. By lactate additions (Table 1) the redox condition in the columns was always kept anaerobic as can be observed from the redox measurements at the inflow and outflow of the sediment column. Besides, some continuous removal of *cis*-DCE, VC and ethene from the column was also observed, most probably due to diffusion through the Teflon tubing (Parker et al. 1990; Rowe et al. 1995). Parker et al. (1990) showed a more than 10 % loss of chlorinated organic in the stored sampling with Teflon material after 8 h. Therefore, a continuous dissipation of CVOCs was observed; however, the formation and development trend of these compounds could still well be observed to determine biodegradation of *cis*-DCE and reflect the DHC resilience. The used tubing was the best to our knowledge among the available material that could be used in this experiment.

**Table 1:** Overview of actions and related additions of different chemicals

Action	Day	Addition of				
		Lactate (225 g/L)	Inoculum (5 mL)	*cis*-DCE (5 mL; 395 mg/L)	NaNO_3_ (5 mL; 85 mg/L)	Other
1	20	1.5 mL				
2	30		1.3 × 10^8^ cells/mL			
3	41		3.2 × 10^8^ cells/mL	x		
4	45	0.5 mL				
5	48			x		
6	51			x		
7	55	0.5 mL				
8	56			x		
9	57				x	
10	62			x		
11	66	1 mL				
12	71	0.5 mL				
13	76			x		
14	80		2.5 × 10^8^ cells/mL			
15	84	0.5 mL				
16	91			x		
17	92	0.5 mL				
18	93		2.9 × 10^8^ cells/mL			
19	94					Nutrient (2.5 mL; Na_2_HPO_4_ 50 g/L, KH_2_PO_4_ 50 g/L, NH_4_Cl 10 g/L)
20	98	0.5 mL		x		
21	101	0.5 mL				
22	104	0.5 mL		x		
23	105				x	
24	114	1 mL				
25	118					NH_4_Cl (2.5 mL; 10 g/L)
26	125		3.6 × 10^8^ cells/mL			
27	132		4.4 × 10^8^ cells/mL			
28	134	0.5 mL				
29	135					Vitamin B_12_ medium (5 mL; 2 mg/L)

#### Experimental procedures

Dynamic redox conditions were simulated by adding lactate as electron donor to achieve lower redox potential or by adding nitrate as electron acceptor to raise the redox potential. In total, two cycles of low and elevated redox potential were performed. During the experiment, liquid was sampled at position 5 in Fig. 1, while headspace sampling was done at the side arms of the buffering bottle. Addition of chemicals was also done via these side arms. The experiment lasted for 140 days with in total of 28 actions which are listed in Table 1.

Important procedures included (1) monitoring initial behaviour of selected aquifer material in the column system in the adaptation period of 20 days; (2) redox conditioning by adding lactate as electron donor to achieve an environmental condition with redox potential around −450 mV to stimulate reductive dechlorination (Ni et al. 2014). For this purpose, three lactate additions on day 20, 66 and 114 were performed with 1.5, 1 and 1 mL lactate stock, respectively (Table 1). Additional lactate additions of 0.5 mL lactate stock on day 45, 55, 71, 84, 92, 98, 101, 104 and 134 were to maintain the low redox potential that is suitable for DHC. The addition of electron donor was found to be necessary as the redox potential slowly increased, due to the continuous diffusion of O_2_ through the Teflon tubing; (3) inoculating the column system with DHC in the period without any chlorinated ethenes present to study the distribution of DHC in the model ATES system (day 30), the subsequent DHC inoculations on day 41, 80, 93, 125 and 132 (Table 1) were performed to start-up or to recover the reductive dechlorination; (4) spiking of *cis*-DCE stock. In total, 9 times of *cis*-DCE spiking with 5 mL *cis*-DCE stock for each; approximately 20 μmol *cis*-DCE/spiking (Table 1) were conducted on day 41, 48, 51, 56, 62, 76, 91, 98 and 104; (5) two times nitrate stock additions (5 mL for each) as redox shock to bring up the redox potential on day 57 and 105; (6) addition of 2.5 mL of nutrient solution containing 50 g/L Na_2_HPO_4_, 50 g/L KH_2_PO_4_ and 10 g/L NH_4_Cl), 2.5 mL of 10 g/L NH_4_Cl and 5 mL vitamin B_12_ medium on day 94, 118 and 135 for promoting the recovery of reductive dechlorination.

## Results

### Redox potential and other redox indicators

**Fig. 2:**
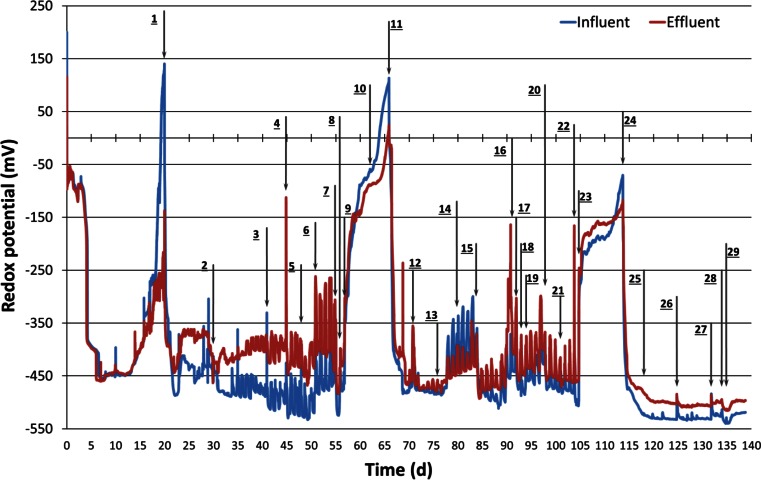
Redox potential of the influent (*blue line*) and the effluent (*red line*). *Black arrows* and with *numbers* indicate different actions listed in Table 1

During the first 20 days, the redox potential in the column was monitored without any redox conditioning steps. After an initial stabilization at −100 mV, the redox potential rapidly dropped to −350 mV on day 4 and further to −450 mV on day 6 (Fig. 2). Thereafter, starting at day 12, the redox potential increased up to about 150 mV on day 20, ascribed to oxygen diffusion through the Teflon tubing.

After addition of lactate (action, day 20), the redox potential in both influent and effluent decreased to −450 mV and remained stable at this low value. This low redox potential is reported to be suitable for reductive dechlorination and methanogenesis (Christensen et al. 2000; ITRC 2005; Ni et al. 2014). The nitrate addition on (action 9, day 57) resulted in an increase of redox potentials up to −150 mV within 2 days and continued to increase to 107 mV in the influent and 25 mV in the effluent during the following 8 days (Fig. 2). The subsequent lowering of the redox potential was initiated by the addition of lactate on day 66 (action 11). Similar to the first lactate addition, the redox potential dropped sharply to below −400 mV within 1 day and remained stable until day 105 when the second nitrate addition was performed (action 23). After this second nitrate addition, the redox potential increased up to −70 mV, which was around 170 mV below the value reached upon the first nitrate addition. Low redox potentials (from −400 to −500 mV) were again obtained after the third addition of lactate on day 114 (action 24). Interestingly, the redox potential showed less noise after this lactate addition compared to earlier additions of lactate (Fig. 2, after action 24).

**Fig. 3:**
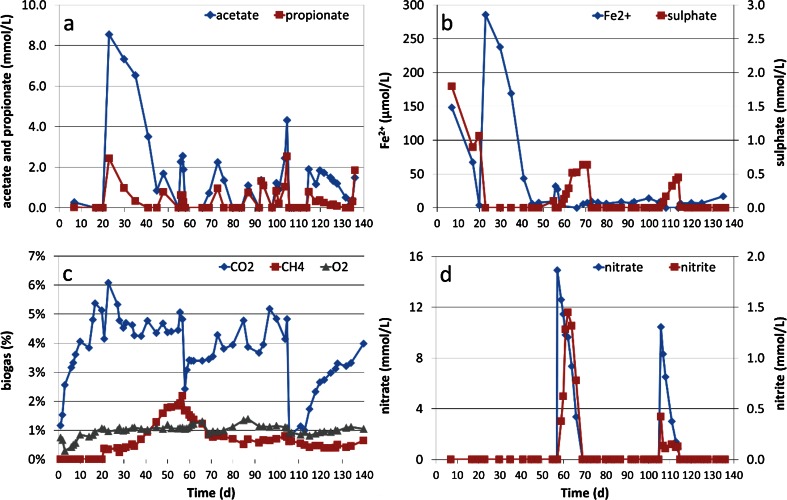
Concentrations of different compounds as a function of experimental time. **a** (*top left*) represents acetate and propionate concentration with unit of mmol/L in liquid sampling port; **b** (*top right*) represents Fe^2+^ (primary *y*-axis) and sulphate (secondary *y*-axis) concentration with unit of μmol/L and mmol/L, respectively, in liquid sampling port; **c** (*bottom left*) represents O_2_, CH_4_ and CO_2_ gas composition in the headspace of the buffering bottle in percentage; **d** (*bottom right*) represents nitrate (primary *y*-axis) and nitrite (secondary *y*-axis) concentration with unit of mmol/L in liquid sampling port

Prior to any addition of lactate, acetate was detected with a maximum concentration of 0.27 mmol/L on day 7 and was consumed before the first lactate addition on day 20. Propionate was not detected at this stage. After the first addition of lactate, the concentration of acetate increased rapidly to 8.55 mmol/L, while the concentration of propionate increased to 2.43 mmol/L (Fig. 3a). The concentration of these two volatile fatty acids (VFAs) followed a pattern of rapid increase and gradual decrease with every lactate addition. After nitrate additions (action 9, day 57 and action 23, day 105), the decrease of VFA concentration was fast, however. Lactate was not detected along the experiment indicating that the production of acetate and propionate resulted from a fast process such as lactate fermentation (Ni et al. 2014; Seeliger et al. 2002).

The initial concentration of Fe^2+^ in the aqueous phase was close to 148 μmol/L and decreased to below 4 μmol/L during the 3 weeks prior to the first addition of lactate (Fig. 3b). The first addition of lactate lead to a large increase of Fe^2+^ concentration up to 286 μmol/L on day 23 whereafter the concentration returned to low values (0–20 μmol/L). Subsequent additions of lactate did not result in elevated Fe^2+^ concentrations (Fig. 3b). Sulphate concentration, (1.8 mmol/L at the start), decreased steadily to about 1.0 mmol/L during the first 20 days. Thereafter, the sulphate concentration declined quickly to negligible zero with first lactate addition (action 1, day 20). Only after nitrate addition (action 9, day 57; action 23, day105), elevated sulphate concentrations were observed (Fig. 3b).

Biogas analysis showed that the concentration of CO_2_ increased immediately at the start of the experiment and the percentage reached a highest value of around 6 % on day 23 (Fig. 3c). In the two periods when nitrate was added (action 9, day 57; action 23, day 105), the CO_2_ percentage rapidly decreased to about 2 and 1 %, respectively. After subsequent lactate addition (action 11, day 66; 24, day 114), the CO_2_ gradually returned to values around 4 %, with some fluctuations in the rest of periods (Fig. 3c). CH_4_ was detected after the first lactate addition (action 1, day 20). The highest CH_4_ concentration was observed right before nitrate addition on day 57 (around 2 %). After the second nitrate addition, the concentration slowly declined to less than 1 % and further below 0.5 %. The O_2_ percentage in the headspace of the buffering bottle was stable at about 1 % during the experiment (Fig. 3c).

Neither nitrate nor nitrate was detected before nitrate addition on day 57. Nitrate concentration peaked at 15.0 mmol/L on day 58, after which the production of nitrite occurred with a maximum concentration of 1.45 mmol/L (Fig. 3d). After lactate addition (action 11, day 66), nitrate and nitrate concentrations rapidly decreased to below detection limits on day 69. A similar pattern was observed after the second nitrate addition although the peak concentrations for both nitrate (10.5 mmol/L) and nitrite (0.4 mmol/L) were lower than those in the first time. The nitrite peak was smaller and occurred simultaneously with nitrate, indicating the presence of adapted nitrate and nitrite reducing microbial community and thus faster denitrification process.

### Reductive dechlorination and microbial population

**Fig. 4:**
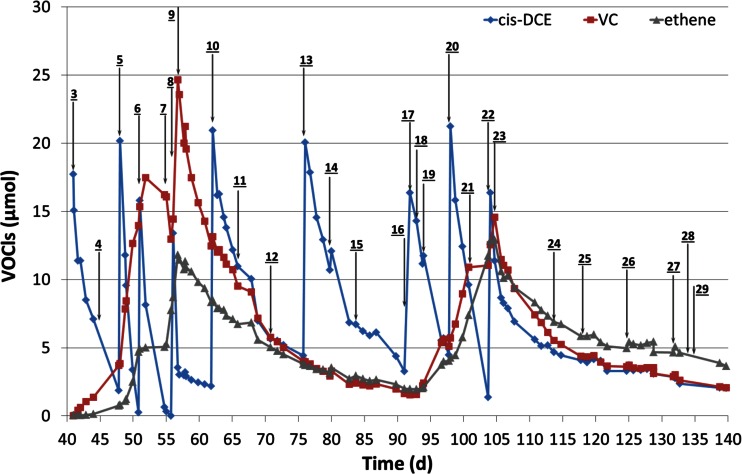
Concentration of *cis*-DCE (*blue diamond*), VC (*red square*) and ethene (grey triangle) as a function of experimental time. *Black arrows* and with *numbers* indicate different actions listed in Table 1

First *cis*-DCE was spiked on day 41 (action 3) when the column remained at stable conditions regarding the redox indicators. Readily after spiking with *cis*-DCE, the presence of VC and ethene was observed, indicating that the complete pathway of reductive dechlorination is occurring (Fig. 4). When the redox went up to −250 mV unexpectedly, the production of VC and ethene ceased (day 52). However, after the lactate addition on day 55 (action 7), the production of these compounds resumed and reached 24.6 and 11.8 μmol for VC and ethene, respectively.

After the first nitrate addition (action 9, day 57), the production of VC and ethene stopped, although all three compounds disappeared with comparable rates from the reactor (Fig. 4), most probably due to diffusion via the via the Teflon tubing. Restoring the redox potential to its previous low values by the addition of lactate (action 11, day 66 and action 12, day 71) did not result in any noticeable recovery of reductive dechlorination. Even after adding fresh DHC (action14, day 80), two additions of lactate (action 15, day 84, action17, day 92), followed by DHC inoculum (action18, day 93 (DHC), dechlorination did not resume. Only after adding nutrients (action 19, day 94), the production of VC and ethene observably increased, which was further enhanced upon an extra addition of *cis*-DCE and lactate (action 20, day 98).

The second addition of nitrate (action 23, day 105) aimed at a repetition to test the resilience of reductive dechlorination after a redox shock. This time, reductive dechlorination as evidenced by production of VC and ethene, did not resume after addition of lactate (action 24, day 114), nutrients (action 25, day 118), and two DHC inoculum (action 26, day 125, action 27, day 132), again lactate addition (action 28, day 134) and finally addition of vitamin B_12_ (action 29, day 135).

**Fig. 5:**
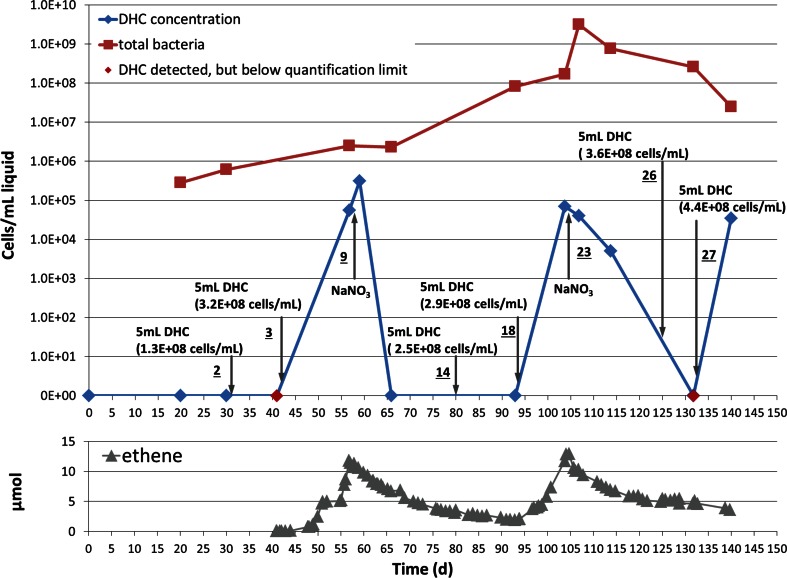
Change of DHC (*blue* or *red diamond*) and total bacteria (*red square*) concentrations in the liquid phase along time and with comparison to the change of ethene concentration (*grey triangle*) during the experiment. *Black arrows* indicate either addition of active DHC inoculum (action 2, 3, 14, 18, 26 and 27) or nitrate (action 9 and 23)

The presence of DHC in the liquid phase followed with time is presented in Fig. 5, as well as the concentration of the total bacteria in the liquid phase. During the first 30 days, DHC concentration was below detection limit. Readily after inoculation with 5 mL DHC (1.3 × 10^8^ cells/mL), the DHC could be detected in liquid but below the quantification limit (red diamond on day 40 in Fig. 5).

After *cis*-DCE was spiked and reductive dechlorination occurred (day 40–56), the DHC concentration increased to 5.6 × 10^4^ cells/mL, which was about 2 % of the total bacteria concentration in the liquid phase. Interestingly, an increased concentration of DHC (3.1 × 10^5^ cells/mL) was detected in the liquid only 2 days after nitrate addition, while reductive dechlorination had ceased. Shortly after this increase, the concentration of DHC diminished to below detection limit in the next analysis on day 66 (Fig. 5) and remained at this low concentration even after a new inoculation (action 14, day 80).

After a second inoculation (action 18, day 93) and the addition of nutrients (action 19, day 94), reductive dechlorination resumed and DHC concentration increased to 6.9 × 10^4^ cells/mL, which was quantifiable though low compared to the amount of total bacteria (1.7 × 10^8^ cells/mL).

The second nitrate addition showed that the DHC concentration gradually declined to 5 × 10^3^ cells/mL after 10 days (Fig. 5) (action 23, day 105). Aiming to resume the reductive dechlorination, DHC was inoculated twice (action 26, day 125, action 27, day 132) resulting in a DHC concentration of 3.5 × 10^4^ cells/mL in the liquid, at the end of experiment. However, reductive dechlorination, as evidenced by an increase in ethene, did not resume.

The total bacteria concentration in the liquid phase with initial concentration of 2.8 × 10^5^ cells/mL significantly expanded during the experiment. The maximum expansion reached four orders of magnitude on day 107 with concentration of 3.2 × 10^9^ cells/mL, despite the final concentration reduced to 2.5 × 10^7^ cells/mL. A variety of microbiological processes might be stimulated by this bacterial community, as was reflected by the responses upon redox chemistry and bioprocess indicators presented in Fig. 3.

## Discussion

### Redox parameters

After the start of the experiment, the redox potential in both inflow and outflow showed a rapid lowering down to −380 mV at day 4, followed by a period of gradual decrease down to −400 mV at day 6 and again a rapid decrease down to −450 mV at day 7. Most probably, organic matter is produced in this phase which released by packing of the column and by organic particles that are disrupted by shear forces at the high liquid flow velocity (Wu et al. 2009). The released organic matter can function as electron donor for biodegradation as we observed in a batch experiment with the same aquifer material at intensive shaking condition (Figure S3). The period of gradual decreasing redox potential between day 4 and 6 probably gave onset to the reduction of readily available Fe^3+^ species to dissolved Fe^2+^, as evidenced by the remaining Fe^2+^ concentration measured at day 7.

The net removal of Fe^2+^ from the liquid phase together with sulphate points towards continued reduction of both iron and sulphate and subsequent precipitation as iron sulphides between day 7 and day 17. Then, however, the gradual increase in redox potential due to the oxygen diffusion inverted these processes as, between day 18 and 20, sulphate partly re-dissolved. The on-going removal of Fe^2+^ from the liquid phase implies that during this period also Fe^2+^ being oxidized and precipitated as iron (hydr)oxides again. With the first lactate addition at day 20, the remaining sulphate was rapidly reduced to sulphide (Fig. 3b). The rapid increase in Fe^2+^ after this first lactate addition, when no DCE had yet been added to the system, seems to be related to the concurrent high acetate concentration (Fig. 3a), which suggests increased iron solubility due to Fe-acetate complexation (Theis and Singer 1974). Thereafter, the Fe^2+^ concentration was always below 30 μmol/L, which can be caused by precipitation with sulphide again or carbonate, leading to formation of Fes and FeCO_3_ with negligible solubility (Bénézeth et al. 2009; Davison 1980).

After 12 days, the readily available organic matter seemed to be depleted and could not donate electrons to sufficiently compensate the inflow of oxygen that was presumed to diffuse through the Teflon tubing into the system (Giacobbe 1992; Peirce 1958). This is supported by the observation that the redox potential of the inflow is continuously higher than the redox potential of the outflow. By addition of lactate, the effect of oxygen diffusion on the redox potential was negligible and a stable low redox potential was obtained.

After the nitrate additions (action 9, day 57 and action 23, day 105), the redox potential elevated to 100 and −70 mV, respectively (Fig. 2). This increase resulted in a temporary increase of sulphate, as sulphide oxidation was occurring coupled with nitrate as terminal electron acceptor (Aminuddin and Nicholas 1973; Brunet and Garcia-Gil 1996). In these nitrate reducing periods, Fe^2+^ as electron donor was also oxidized by nitrate leading to no detectable concentration in the dissolved phase (Straub et al. 1996). The nitrate is then reduced to nitrite by lactate or VFAs (Fig. 3d) which is consistent with other studies (Elefsiniotis et al. 2004; Fass et al. 1994).

The build-up of both CO_2_ and CH_4_ in the gas phase, especially in the first period of biostimulation, is the evidence of methanogenic redox conditions being achieved. The sudden decrease in CO_2_ concentration in the headspace upon nitrate addition was attributed to the fast consumption of protons and electrons in the system for nitrate reduction (Guerrero et al. 1981; Postma et al. 1991). The consumption of protons led to a clear increase in pH level up to 7.6 and 8.0 (Figure S4, influent), resulting in of the uptake of CO_2_ (g) into HCO_3_^−^ (aq) (Stirling 2011). The decrease of methane concentration upon nitrate addition is attributed to the inhibition of methanogenesis by nitrate and diffusion of methane through the Teflon tubing (Božic et al. 2009; Roy and Conrad 1999).

In summary, the behaviour of redox potential properly reflected other microbiological processes, such as fermentation, Fe(III) and sulphate reduction, methanogenesis and denitrification. These processes are considered to be important indications on reductive dechlorination (Aulenta et al. 2006; Boopathy 2000; ITRC 2008; Nelson et al. 2002). Therefore, monitoring redox potential in the groundwater is of importance to evaluate the potential and the progress of reductive dechlorination in the subsurface (Ni et al. 2014).

### Resilience of *Dehalococcoides* and robustness of the reductive dechlorination process upon redox changes

At the start of the experiment, DHC was not detected which was expected as CVOCs were absent in the original samples and the redox conditions were unfavourable for reductive dechlorination (Kouznetsova et al. 2010; McCarty 1997; Ni et al. 2014; van der Zaan et al. 2010). The absence of DHC in the original sample is furthermore supported by Cupples et al. (2004) who stated that the decay rate of DHC increases if the concentration of *cis*-DCE and VC as electron acceptor is less than 0.7 μmol. Only after inoculation with 5 mL inoculum of around 10^8^ cells/mL, DHC could be detected, although at a low level. This indicates that if all or most DHC was suspended, its concentration should have been detected as about 10^6^ to 10^7^ cells/mL, based on the total liquid volume (approximately 250 mL) within the column. In fact, during the whole experiment, the observed DHC concentration by sampling from the liquid phase was virtually negligible compared to the DHC that was inoculated. Even the quantifiable measurements observed in periods of active dechlorination showed that DHC concentrations were still less than 0.1 % of the previously added amount, indicating that large part of DHC is preferentially attached to the soil matrix. This finding is consistent with other studies which show DHC are less planktonic and mostly favour to attach to soil (Amos et al. 2009; Behrens et al. 2008). Besides, Doong et al. (1997) showed the performance of dechlorinating bacteria is 2 to 5 times better with attachment to porous medium. Furthermore, growth of DHC could be expected in the two active periods of reductive dechlorination (Cupples et al. 2003; He et al. 2003; Scheutz et al. 2008), which could be the reason that quantifiable DHC were detected in the liquid phase on day 56 and 104. The small (one order of magnitude) increase in DHC concentration between day 57 and 59, with the subsequent measurement below detection, could be due to interruption by nitrate of DHC activity, leading to its detachment and transfer to the liquid phase with DHC dying off eventually. The balance between the rate of DHC detachment and DHC decay after the second nitrate dose seems slightly different. Interestingly, the final analysis showed a quantifiable DHC concentration in the liquid but without observable dechlorination proceeding. An explanation may be *cis*-DCE concentration, which was below 5 μmol after day 115 (Fig. 4), was too low for DHC to metabolize or even survive, leading to some persisting inoculated cells to be observed in the liquid.

The inhibition of nitrate on reductive dechlorination found in this study upon nitrate addition was in line with previous research (Nelson et al. 2002; Ritter et al. 2003; Schlicker et al. 2000). After the first nitrate shock, the reductive dechlorination process could be restarted, but only by adding new DHC and nutrients. After the second nitrate shock, no restart of the dechlorination process at any significant rate could be observed. Irreversible inhibition of dechlorination by elevated redox potential has been reported by several studies (Amos et al. 2008; Doğan-Subaşı et al. 2013; Nelson et al. 2002). Yet, recovery of reductive dechlorination after applying chemical oxidation by permanganate and persulphate followed by bioaugmentation in laboratory experiments has also been described (Doğan-Subaşı et al. 2013; Hrapovic et al. 2005; Sahl et al. 2007). In a field test, recovery was even reported to occur without bioaugmentation but ascribed to inflow of groundwater and recolonisation by microorganisms from unaffected zones (Sutton et al. 2015). Although these studies are confronted with lag periods of up to hundreds of days, recovery of reductive dechlorination is proven with amendments which were mainly attributed to bioaugmentation. In contrast, our column was much less robust, even after several amendments including sufficient electron donor, nutrient, bioaugmentation and vitamin B_12_ which are beneficial to either DHC growth or reductive dechlorination (He et al. 2007; Hu et al. 2013; ITRC 2005; Miura et al. 2015; Reinhold et al. 2012). One reason could be the much higher flow rates in our experiment compared to other column systems (Hrapovic et al. 2005; Sahl et al. 2007). Another explanation could be that the diffusion rate of *cis*-DCE, VC and ethene overcame the dechlorination rate, concealing any ongoing dechlorination process from being observed. The last *cis*-DCE spiking was performed on day 104 and its concentration had dropped to below 5 μmol after 10 days (Fig. 4). Such low *cis*-DCE concentration might be insufficient to show measurable conversion into VC or ethene by DHC, while diffusion was always proceeding.

### Implications for combining CVOCs bioremediation and ATES

In this novel study, we fundamentally investigated the impact of ATES on enhanced CVOCs bioremediation by addressing many important microbiological processes that occur in both systems. The findings are relevant and meaningful to the combination of ATES and enhanced bioremediation. Our results show that reductive dechlorination is sensitive to increased redox potentials. Therefore, proper operation of ATES is recommended avoiding the mixing of oxidized groundwater into more reduced aquifers. Such an approach is already in practise for iron rich groundwater, as iron precipitates and therefore clogging will occur with oxygenated water. The heterogeneity of the subsurface would also provide some protection of microorganisms against negative disturbances, for example, by providing stabile redox micro niches in the subsurface. Hence, the risk of interruption of dehalogenation by oxidized water in practise is expected to be less than that is shown in our study. Further, it was shown that at the mimicked ATES conditions, active dehalogenating DHC cells appear to be preferentially attached to the soil matrix, instead of being mobile and transported with the water phase. Hence, for the combination ATES and bioremediation of CVOCs, bioaugmentation by injecting mobile DHC will profit from this behaviour, as it can be expected that DHC augmentation will lead to attachment to the soil matrix after injection. From our experiment it is expected that inoculation with DHC in the warm well creates optimal conditions in the warm ATES well for reductive dechlorination at high biomass concentrations and at elevated temperature. These results show that practical application of the combination of ATES and in situ dehalogenation technologies can be promising by proper engineering, initial characterization, continued monitoring of the groundwater systems and well-designed bioaugmentation procedures.

## Electronic supplementary material

ESM 1 (PDF 627 kb)
