# Supplementary material for: Combination of aquifer thermal energy storage and enhanced bioremediation: resilience of reductive dechlorination to redox changes
Source: Appl Microbiol Biotechnol. 2015 Dec 28;100:3767–80. doi: 10.1007/s00253-015-7241-6 (PMC4803826; doi:10.1007/s00253-015-7241-6)
Supplement: Supplementary file 1 — (PDF 627 kb) [file 253_2015_7241_MOESM1_ESM.pdf]

Applied Microbiology and Biotechnology

Combination of Aquifer Thermal Energy Storage and Enhanced Bioremediation: Resilience of Reductive Dechlorination to Redox Changes

Zhuobiao Ni [a,b,*], Pauline van Gaans [c], Martijn Smit [a,d], Huub Rijnaarts [a], Tim Grotenhuis [a]

[a] Sub-Department of Environmental Technology, Wageningen University, P.O. Box 17, 6700 AA Wageningen, the Netherlands

[b] Wetsus, Eoropean Centre of Excellence for Sustainable Water Technology, P.O. Box 1113, 8900 CC Leeuwarden, the Netherlands

[c] Soil and Groundwater Systems, Deltares, P.O. Box 85467, 3508 AL Utrecht, the Netherlands

[d] Present address: Eurofins Analytico, P.O. Box 459, 3770 AL Barneveld, the Netherlands

* Corresponding author: nizhuobiao@hotmail.com. Tel: +31647465817

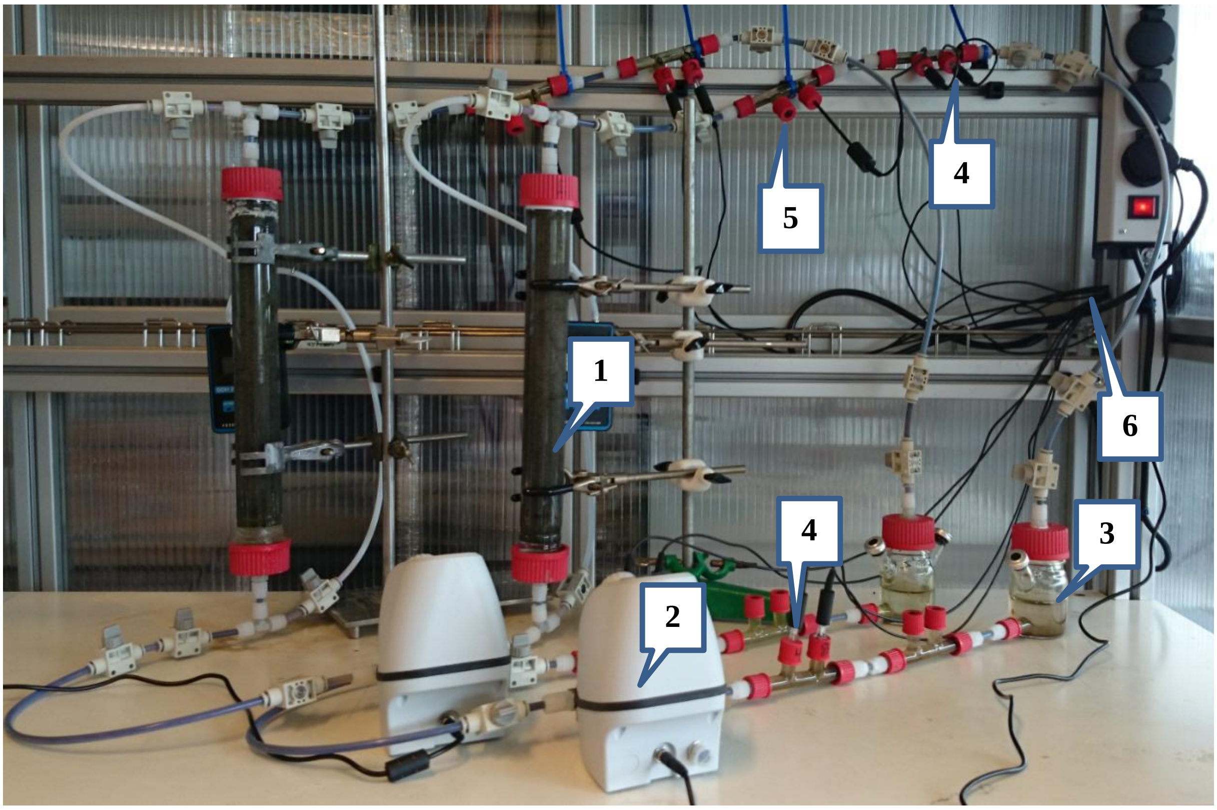

Figure S1 Photo of the column set-up. 1: sediment column with glass filters on the top and bottom; 2: membrane pump; 3: buffering bottle; 4: redox and pH electrodes 5: liquid sampling port; 6: electrode wires connected to computer (not in this picture).

Table S1 Specification of column set-up.

Parameters	Value	Unit
Length	30.0	[cm]
Diameter	3.6	[cm]
Wet weight of aquifer material	625	[g]
Porosity	0.3	[-]
Pore volume	92	[mL]
Hydraulic Retention Time (HRT)	9.2	[min]
Volume of tubes and other connection parts	100	[mL]
Total liquid volume	240	[mL]

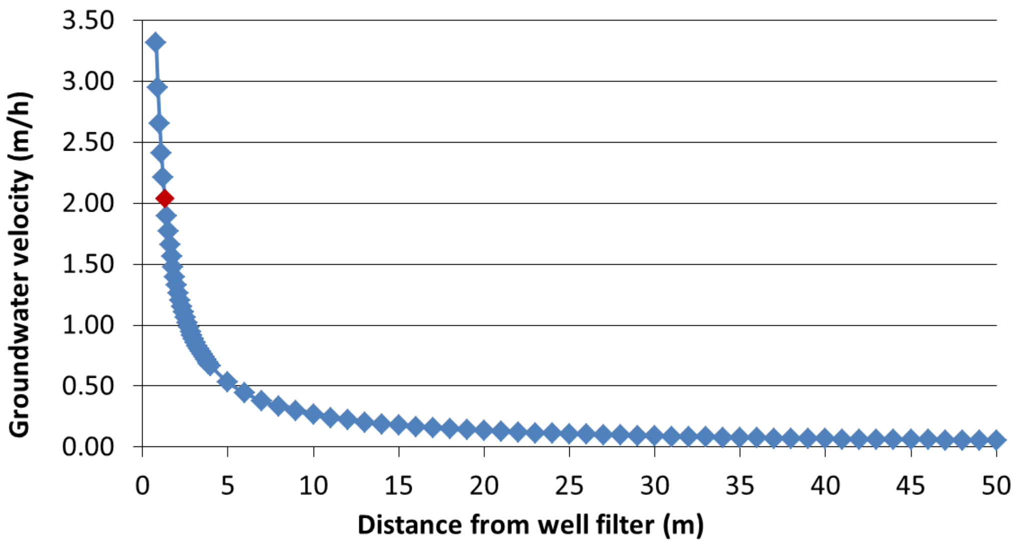

Figure S2 Groundwater velocity as a function of distance from well filter in ATES subsurface. The red diamond represents the mimicked water velocity in our column study.

This ATES operates with a flow rate of 100 m^3/h (Q) and with 20 m filter length (L). Using an aquifer porosity of 0.3 (ε), the relation between groundwater velocity (v) and distance from well filter (x) can be calculated via the following equation: $v = \frac{Q}{2\pi x L\varepsilon}$

Therefore, the groundwater velocity is 2 m/h when it is 1.3 m from the well filter. A flow rate of 10 mL/min was applied in our column experiment, with the parameter given in Table S4.1, the water velocity in the column is calculated to be 2 m/h (flow rate over cross-section area and porosity).

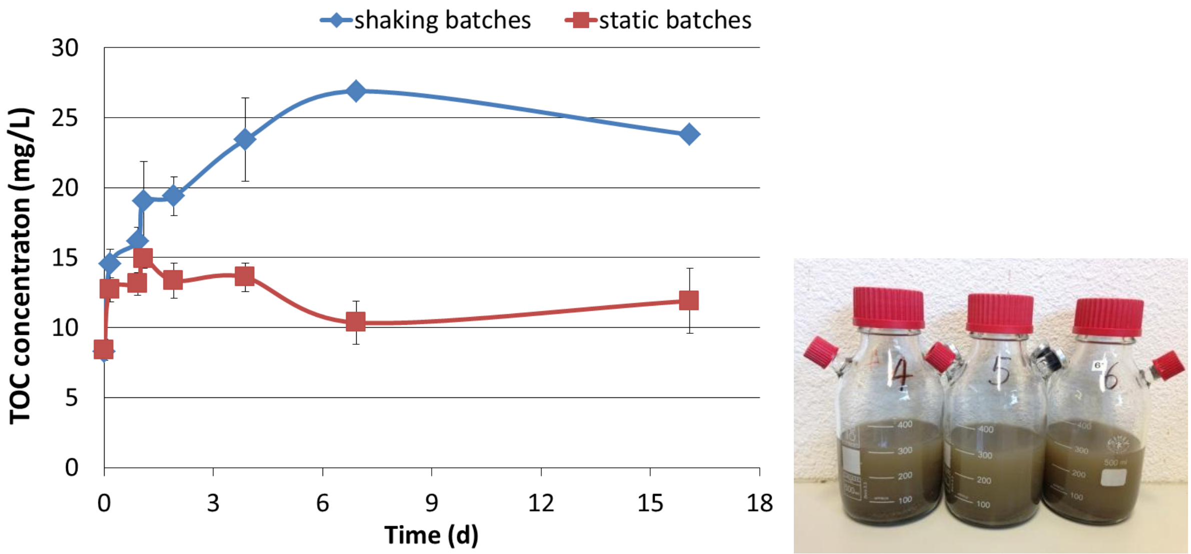

Figure S3 TOC (total organic carbon) concentration as a function of experimental time (left) in shaking (blue diamond) and static (red square) conditions in preliminary test upon effect of shaking on release of organic carbon at 25 ˚C and photo of shaking batches (right). Error bars represent standard deviation of the triplicates. When they are invisible, they are smaller than the symbols and hidden behind.

The preliminary experiment was performed using 500 mL double-side armed which contained 100 g wet aquifer material and 250 mL anaerobic tap water, with anaerobic headspace of 99% N_2 and 1% CO_2. One group of batches were shaken orbitally at a speed of 175 rpm, while other group of batches were always static during the experiment. The experiment was done in triplicate and at 25 ˚C. Liquid was periodically sampled for TOC measurements. TOC concentration was determined was measured by Hach Lange cuvette tests with Dr Lange LCK-385 (3-30 mg/L) on a Xion 500 spectrophotometer.

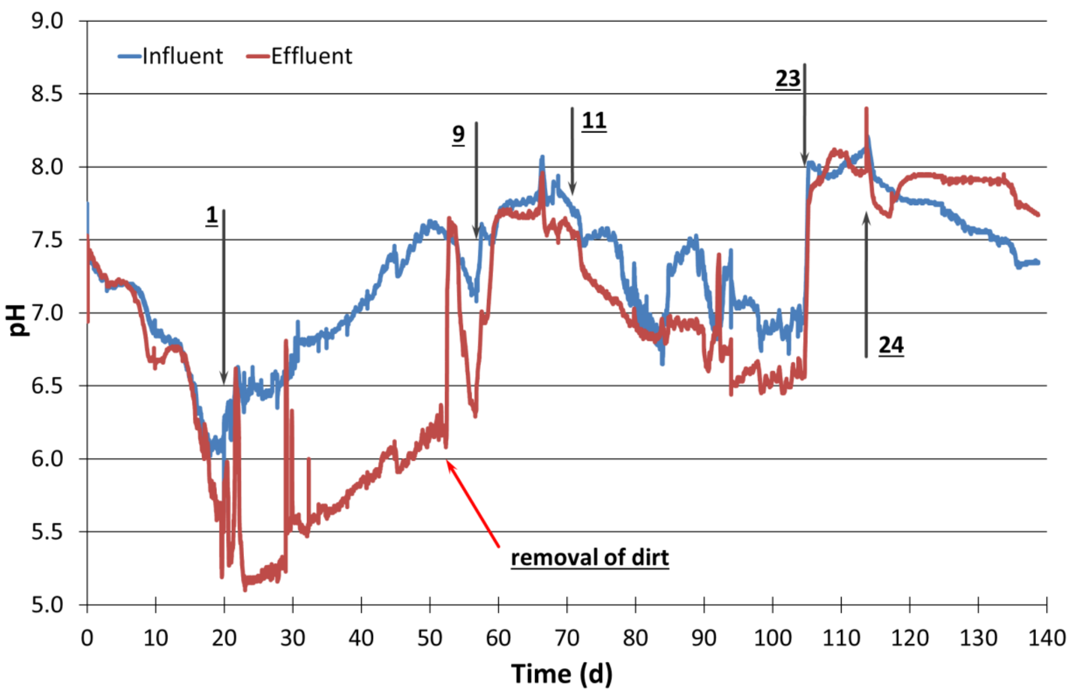

Figure S4 The pH of the influent (blue line) and the effluent (red line). Black arrows and with numbers indicate different actions listed in Table 4.1. Red arrow stands for the removal of dirt around the effluent pH electrode.

A relatively large deviation between pH readings of the influent and effluent was recorded from around day 23. This deviation lasted until day 52, when some dirt around the effluent pH electrode was observed. With removal of the dirt, pH readings of the influent and effluent were consistent thereafter.
